# Rheological Properties of Small-Molecular Liquids at High Shear Strain Rates

**DOI:** 10.3390/polym15092166

**Published:** 2023-05-02

**Authors:** Wenhui Li, JCS Kadupitiya, Vikram Jadhao

**Affiliations:** Intelligent Systems Engineering, Indiana University, Bloomington, IN 47408, USA; wli11@iu.edu (W.L.); kadu@iu.edu (J.K.)

**Keywords:** shear thinning, molecular orientation, nonequilibrium molecular dynamics simulations, machine learning, principal component analysis, elastohydrodynamic lubrication

## Abstract

Molecular-scale understanding of rheological properties of small-molecular liquids and polymers is critical to optimizing their performance in practical applications such as lubrication and hydraulic fracking. We combine nonequilibrium molecular dynamics simulations with two unsupervised machine learning methods: principal component analysis (PCA) and t-distributed stochastic neighbor embedding (t-SNE), to extract the correlation between the rheological properties and molecular structure of squalane sheared at high strain rates ($10^{6}$–$10^{10}$$s^{-1}$) for which substantial shear thinning is observed under pressures P∈0.1–955 MPa at 293 K. Intramolecular atom pair orientation tensors of 435×6 dimensions and the intermolecular atom pair orientation tensors of 61×6 dimensions are reduced and visualized using PCA and t-SNE to assess the changes in the orientation order during the shear thinning of squalane. Dimension reduction of intramolecular orientation tensors at low pressures P=0.1,100 MPa reveals a strong correlation between changes in strain rate and the orientation of the side-backbone atom pairs, end-backbone atom pairs, short backbone-backbone atom pairs, and long backbone-backbone atom pairs associated with a squalane molecule. At high pressures P≥400 MPa, the orientation tensors are better classified by these different pair types rather than strain rate, signaling an overall limited evolution of intramolecular orientation with changes in strain rate. Dimension reduction also finds no clear evidence of the link between shear thinning at high pressures and changes in the intermolecular orientation. The alignment of squalane molecules is found to be saturated over the entire range of rates during which squalane exhibits substantial shear thinning at high pressures.

## 1. Introduction

Rheological properties of small-molecular and polymeric liquids are critical to optimizing their performance in applications such as lubrication and hydraulic fracking [1,2,3]. For example, shear thinning of lubricants operating under elastohydrodynamic lubrication conditions of high strain rates (>$10^{5}$ $s^{-1}$) and high pressures (>0.5 GPa) lowers friction between mechanical contacts. However, a substantial reduction of viscosity with increasing shear strain rate may cause the lubricant to squeeze out, leading to wear of the mechanical contacts [1,4,5]. Similarly, in hydraulic fracking applications, the Newtonian viscosity and shear thinning of fracturing fluids determine whether the fluid viscosity is sufficiently large to reduce particle settling rates and sustain fractures of desired geometry against closure stresses imposed by the reservoir [3,6].

Experimental advances have enabled the design of rheometers that can now exceed 1 GPa at $10^{4}$ $s^{-1}$ and reach shear stresses of up to ∼30 MPa [7,8]. However, the experimental measurements of rheological properties (e.g., viscosity) at high rates may be affected by increases of temperature during shear [1]. Much higher shear stresses (∼150 MPa) can be reached in experiments such as those on idealized elastohydrodynamic lubrication contacts sheared under strain rates in real devices [1,5], but these experiments measure an average stress over contact regions with a range of local strain rates, pressures, and temperatures [4,9].

In the last several decades, thanks to the increased computational power and development of reliable atomistic models, nonequilibrium molecular dynamics (NEMD) simulations have emerged as a powerful tool to extract macroscopic rheological properties of liquids sheared at high strain rates that are difficult to reach in experiments [10,11,12,13,14,15,16,17,18,19,20,21]. NEMD simulations have been used to evaluate the rheological properties of polymer solutions with long-chain molecules (e.g., polyethylene melts) [19,20,21] and small-molecular liquids such as the short-chain alkanes in the $C_{20}$–$C_{40}$ mass range [10,12,14,16,17,18,22], which are the main constituents of synthetic and mineral based lubricant basestocks. In addition to macroscopic rheological properties, these simulations produce molecular scale information in the form of large, high-dimensional datasets. The analysis of these datasets via conventional post-processing approaches is time consuming, which often leads to under-utilization of the information generated in simulations. This constitutes a key limiting factor in exploiting the NEMD approach to deeply interrogate the microscopic origins of the macroscopic flow behavior. We illustrate this challenge with the case of NEMD simulations of squalane ($C_{30}H_{62}$), a short-chain alkane that has been widely used as a model elastohydrodynamic lubrication fluid.

Using a united-atom model [23], we performed NEMD simulations of squalane for strain rates $10^{5}$–$10^{10}$$s^{-1}$ in order to examine the nature of its shear thinning at temperature T=293 K as the pressure increases from 0.1 to 1200 MPa [17,18,24]. The shear viscosity was observed to decrease with increasing strain rate across different pressures. The orientation of the end-to-end molecular vector along the shear and velocity gradient directions was extracted and observed to change with strain rate at low pressures (e.g., 0.1, 100 MPa) but was saturated for all strain rates $\dot{\gamma }>10^{6}$$s^{-1}$ at high pressures (e.g., 636, 875 MPa). A more informative link between rheological properties and changes in the molecular structure can be established by examining the intramolecular orientation tensors associated with not just the end-to-end atom pair, but all the 435 distinct atom pairs associated with a squalane molecule. Further, the orientation tensor is a 3 × 3 matrix, containing 6 non-trivial components which includes the component along the vorticity direction and the off-diagonal elements. Analyzing this 435×6 dimensional information is difficult using conventional postprocessing tools, which inhibits a deeper interrogation of the link between molecular-scale features and rheology.

In a recent paper [24], we introduced an approach that combined NEMD simulations and machine learning (ML) methods to analyze the information embedded in these high-dimensional datasets. The approach was illustrated by using dimension reduction methods such as principal component analysis (PCA) [25] and t-distributed stochastic neighbor embedding (t-SNE) [26] to reduce and visualize the information encoded in the 435×6 dimensional data describing the intramolecular orientation tensors associated with the shear flow of squalane. ML expedited the analysis of the intramolecular orientation data and revealed the competing roles of the end-to-end atom pairs and the side atom pairs to the variations in the intramolecular orientation tensor components at low pressures (0.1 and 100 MPa). At high pressures, the rearrangement of the side atom pairs dominated the variations in the tensor components.

In this paper, we build on our earlier introductory work [24] and extend the NEMD-ML approach to perform an in-depth study of the link between shear thinning of squalane and changes in the associated molecular structure. PCA and t-SNE are used to analyze the contributions of the side-backbone atom pairs, end-backbone atom pairs, short backbone-backbone atom pairs, and long backbone-backbone atom pairs to changes in the intramolecular orientation at different pressures. Dimension reduction using PCA of intramolecular orientation tensors at low pressures P=0.1,100 MPa shows that the datasets are best classified by shear rate, revealing a strong correlation between changes in strain rate and the orientation of the side-backbone, end-backbone, short backbone-backbone, and long backbone-backbone atom pairs. At high pressures P≥400 MPa, the orientation tensors are best classified by the pair type (side-backbone, end-backbone, short backbone-backbone, long backbone-backbone) rather than strain rate, which signals an overall limited evolution of intramolecular orientation with changes in strain rate. A closer examination of the long backbone-backbone pairs reveals that the backbone-backbone atom pairs separated by more than 16 bonds can group the orientation tensors into clusters well-separated by strain rate, however, the organization of these clusters does not show any significant correlation with changes in strain rate. The PCA findings are corroborated by t-SNE results. Moving beyond the single-molecule analysis, the influence of intermolecular effects on shear thinning is assessed by extracting orientation tensors associated with atoms belonging to a pair of different squalane molecules. Dimension reduction of the intermolecular pair orientation tensors using PCA and t-SNE reveals a similar contrast between the molecular structure evolution during shear thinning at low pressures vs. that at high pressures. Overall, no clear evidence of the link between shear thinning at high pressures and changes in the intramolecular or intermolecular orientation is observed. Over the entire range of rates during which substantial shear thinning is observed at high pressures, the alignment of squalane molecules is saturated: the intramolecular alignment angle is ∼$10^{{}^{\circ}}$ with respect to the flow direction, and the angle between neighboring squalane molecules is ∼$30^{{}^{\circ}}$.

We note that following our earlier work [24], similar efforts combining NEMD simulations and ML in the area of rheology have appeared in the literature [27,28]. In particular, in Ref. [27] the viscosity-rate scaling of four types of lubricants (spherical, linear, long-branched, and short-branched molecules) confined in mica slit pores was analyzed using NEMD simulations and unsupervised ML to extract several dynamical quantities via an ensemble of trajectories generated from short simulations. Unlike our focus, which is to probe the molecular-scale origins of rheological properties, other studies have focused on the development of ML-based viscosity-prediction models trained on databases of experimental viscosity measurements under different thermodynamic conditions (e.g., temperature, pressure, etc.) [29,30,31,32,33,34,35]. We also note another recent work with a similar goal that explores the design of “digital rheometers”, which can serve as “surrogates” for physical instruments in characterizing the rheological properties of complex fluids [36].

In the next section, we present the molecular model and methods, discussing details not included in our earlier papers, such as the molecular force field parameters, SLLOD algorithm, and the basics of PCA and t-SNE methods. Section 3 presents the results for rheological properties, single atom-pair analysis and dimension reduction of intramolecular orientation tensors, and the results for the analysis of molecular structure based on intermolecular orientation tensors. The final section presents conclusions.

## 2. Models and Methods

In this section, we discuss the molecular model and associated force field parameters used to describe squalane, the NEMD simulation method including the SLLOD algorithm [37,38,39] used to shear the system, and the dimension reduction methods, PCA [25] and t-SNE [40], used to analyze and visualize the high-dimensional simulation output data.

### 2.1. Molecular Model

**Figure 1:**
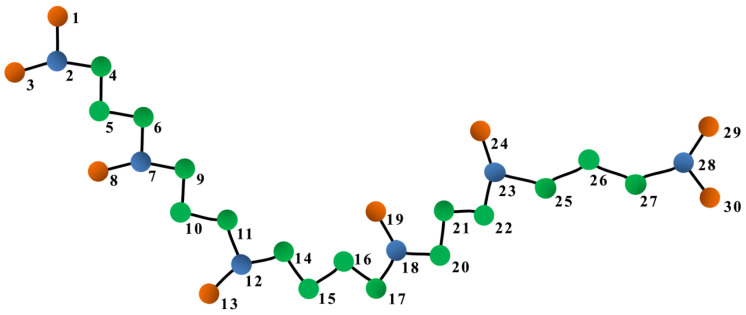
Sketch of a squalane molecule represented by a united atom model, where hydrogen atom(s) and the bonded carbon atom are lumped together. Orange, green, and blue spheres are ${\mathrm{CH}}_{3}$, ${\mathrm{CH}}_{2}$, and CH, respectively. Atoms are labeled with indices to facilitate the identification of atom pairs.

Squalane ($C_{30}H_{62}$) is a branched alkane with a $C_{24}$ backbone and six methyl side branches, as illustrated in Figure 1. Squalane is modeled using a united-atom model developed by Mondello and Grest [23,41], wherein the hydrogen atoms are fused together with the much larger carbon atoms, resulting in three types of united-atoms: ${\mathrm{CH}}_{3},{\mathrm{CH}}_{2}$, and CH. The interactions between these atoms are divided into two categories: non-bonded and bonded interactions. Non-bonded interactions include Coulomb interactions, which are ignored because the united-atoms are charge neutral, and van der Waals interactions, which are described with a pairwise-additive 12-6 Lennard-Jones (LJ) potential:

(1) $$U_{\mathrm{non}-\mathrm{bonded}}\left(r_{ij}\right)=4\epsilon \left[{\left(\frac{\sigma }{r_{ij}}\right)}^{12}-{\left(\frac{\sigma }{r_{ij}}\right)}^{6}\right],$$

where ϵ and σ are the LJ energy depth and size parameters, respectively, and $r_{ij}$ is the distance between atoms *i* and *j*. Table 1 shows the ϵ and σ values used to model LJ interactions between united-atoms of the same type (e.g., ${\mathrm{CH}}_{3}$ and ${\mathrm{CH}}_{3}$). The LJ parameters for interactions between atoms of different types (e.g., ${\mathrm{CH}}_{3}$ and ${\mathrm{CH}}_{2}$) are obtained by using the Lorentz-Berthelot mixing rules: $\sigma_{AB}=\left(\sigma_{AA}+\sigma_{BB}\right)/2$, $\epsilon_{AB}=\sqrt{\epsilon_{AA}\epsilon_{BB}}$, where *A* and *B* denote different types of united atoms. The LJ interactions are applied to all pairs of atoms associated with different squalane molecules, and to those atom pairs that are separated by more than 3 bonds within the same molecule. In all cases, the LJ interactions are truncated and shifted at a cutoff distance of $r_{\mathrm{cut}}=10$ Å.

The interactions among atoms within the same molecule that are separated by less than or equal to 3 bonds are described by bonded interactions, which include a bond stretching potential, a bond angle bending potential, a dihedral potential, and an improper dihedral potential:

(2) $$U_{\mathrm{bonded}}=U_{\mathrm{bond}}+U_{\mathrm{angle}}+U_{\mathrm{dihedral}}+U_{\mathrm{improper}}.$$

The bond stretching between two connected atoms *i* and *j* is represented by a harmonic potential:

(3) $$U_{\mathrm{bond}}\left(r_{ij}\right)=K_{b}{\left(r_{ij}-r_{ij}^{0}\right)}^{2},$$

where $K_{b}=448.126$ kcal/mol/${Å}^{2}$ is a constant, $r_{ij}$ is the bond length, and $r_{ij}^{0}=1.54$Å is the equilibrium bond length. Mondello and Grest used rigid bonds with a fixed bond length [23,41]; we use flexible bonds following Ref. [12].

The angle bending potential associated with a triplet of atoms ijk is also represented by a harmonic potential:

(4) $$U_{\mathrm{angle}}\left(\theta_{ijk}\right)=K_{a}{\left(\theta_{ijk}-\theta_{ijk}^{0}\right)}^{2},$$

where $K_{a}=62.14$ kcal/mol/${\mathrm{rad}}^{2}$ is a constant, $\theta_{ijk}$ is the angle formed by atoms *i*, *j*, and *k* that are connected in sequence (with atom *j* in the middle), and $\theta_{ijk}^{0}=114^{{}^{\circ}}$ is the equilibrium angle.

The dihedral potential is represented as a sum of cosine terms:

(5) $$U_{\mathrm{dihedral}}\left(\phi_{ijkl}\right)=\sum_{n=1}^{5}A_{n}{\left[\cos(\phi_{ijkl})\right]}^{n-1},$$

where atoms *i*, *j*, *k* and *l* are connected in sequence (e.g., atoms 4, 5, 6, and 7 in Figure 1). $\phi_{ijkl}$ is the dihedral angle between the plane formed by atoms *i*, *j*, *k* and the plane formed by atoms *j*, *k*, *l*. $A_{n}$ are constants with units of kcal/mol. There are two types of dihedral angles depending on the type of the two central atoms (*j* and *k*), and Table 2 provides the associated $A_{n}$ values. Note that the signs of $A_{2}$ and $A_{4}$ are incorrect in the original papers [23,41].

The improper dihedral potential is described by a harmonic potential:

(6) $$U_{\mathrm{improper}}\left(\chi_{ijkl}\right)=K_{im}{\left(\chi_{ijkl}-\chi_{ijkl}^{0}\right)}^{2},$$

where in the quadruplet of atoms ijkl, atom *i* is connected to atoms *j*, *k* and *l* (e.g., atoms 2, 1, 3, and 4 in Figure 1). $\chi_{ijkl}$ is the improper dihedral angle between the plane formed by atoms *i*, *j*, *k* and the plane formed by atoms *j*, *k*, *l*. This angle is a measure of how far out of plane atom *i* is with respect to the other three atoms. $K_{im}=40$ kcal/mol/${\mathrm{rad}}^{2}$ is a constant, and $\chi_{ijkl}^{0}=27.25^{{}^{\circ}}$ is the equilibrated angle.

### 2.2. Simulation Methods

All simulations are conducted using the Large-scale Atomic/Molecular Massively Parallel Simulator (LAMMPS) package [42]. First, equilibrium MD simulations of 125 squalane molecules in a cubic simulation box are used to generate samples of squalane under different thermodynamic conditions. The box length is approximately 45 Å, and exhibits a small variation with pressure *P* which changes from 0.1 MPa to 955 MPa and is controlled using a Nose-Hoover barostat [43,44]. Temperature *T* is set to 293 K using a Nose-Hoover thermostat [43,44]. Equilibrium MD simulations are run long enough to equilibrate the system in an NPT ensemble (i.e., fixed number of atoms, pressure, and temperature). Shear is applied afterwards to the equilibrated squalane samples in an NVT ensemble (i.e., fixed number of atoms, volume, and temperature) using the SLLOD algorithm (Section 2.3). Shear rate $\dot{\gamma }$ changes from $10^{6}$ to $10^{10}$ $s^{-1}$. NEMD simulations of shear flow at each shear rate are run for sufficiently long time to ensure that the system reaches steady state and is sheared long enough to generate converged results for shear stress, viscosity, and the structural quantities (e.g., molecular orientation tensors). Generally, lower rates require longer simulation time (which scales as $1/\dot{\gamma }$) to converge. In all cases (equilibrium MD and NEMD simulations), the equations of motion are integrated using Verlet algorithm [45] with a time step of 1 fs. More details can be found in our previous papers [17,18].

The last 1000 frames (time samples) of the molecular trajectory data in the steady-state region are used for extracting the intramolecular and intermolecular orientation tensors between atoms *i* and *j*. Intramolecular orientation tensors are computed for atoms *i* and *j* (j≠i) belonging to the same molecule, while intermolecular orientation tensors are computed for atoms *i* and *j* belonging to different molecules. The orientation tensor $S_{\alpha \beta }^{p}$ with respect to Cartesian coordinates α and β (α, β = *x*, *y*, or *z*) for atom pair *p* (formed by atoms *i* and *j*) is computed as [17,18]

(7) $$S_{\alpha \beta }^{p}=\frac{3}{2}\langle u_{p\alpha }u_{p\beta }\rangle -\frac{1}{2}\delta \left(\alpha ,\beta \right),$$

where $u_{p\alpha }$ is the inner product of the unit distance vector between the atoms associated with atom pair *p* and the unit vector associated with the Cartesian coordinate α. $u_{p\alpha }$ is equivalent to computing cosθ, where θ is the angle between the atom pair vector and the Cartesian coordinate α. $u_{p\beta }$ is a similar quantity computed using the Cartesian coordinate β instead of α. For intramolecular tensors, $\langle \ldots \rangle$ is the ensemble average taken over the total number of molecules and time samples. For intermolecular tensors, $\langle \ldots \rangle$ is the ensemble average taken over time samples and the number of atom pairs belonging to different squalane molecules that are associated with a given distance interval. δ(α,β) is the Dirac delta function, which is 0 for α≠β, and 1 for α=β.

### 2.3. SLLOD

The shear via a fictitious external force is imposed in the x–direction using the SLLOD method [37,38,39,46]. The velocity $v_{x}$ has a gradient along the y–direction, which is defined as the shear rate $\dot{\gamma }=\delta v_{x}/\delta y$. There are many different versions of the SLLOD method (e.g., original SLLOD [37,38,39], g-SLLOD [47], p-SLLOD [48]), all of which yield consistent results for the planar shear considered in our work. The equations of motion associated with the original SLLOD algorithm are

(8) $$\begin{array}{cc} \frac{dr_{i}}{dt} & =\frac{p_{i}}{m_{i}}+r_{i}\cdot \nabla u; \end{array}$$

(9) $$\begin{array}{cc} \frac{dp_{i}}{dt} & =F_{i}^{\phi }+p_{i}\cdot \nabla u, \end{array}$$

where $r_{i}$, $p_{i}$, and $m_{i}$ are, respectively, the position, momentum, and mass of particle *i*, and $F_{i}^{\phi }$ is the total force on particle *i* by other particles derived from the total potential energy, and ∇u is the velocity gradient tensor. The derivation and other details associated with the SLLOD algorithm can be accessed elsewhere [37,38,39,47,48]. Here, we examine closely the SLLOD implementation details in LAMMPS, which have implications in calculating the distances between particles, and thus the orientation tensors introduced above.

**Figure 2:**
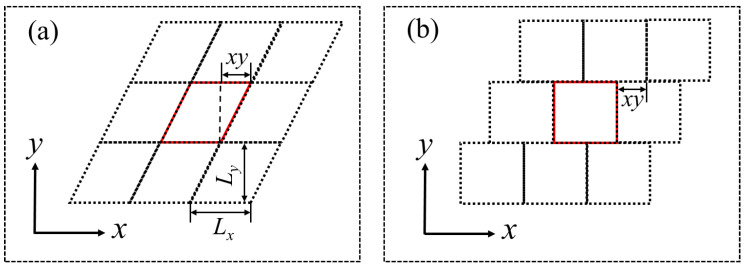
A two-dimensional sketch of periodic boundary conditions (PBCs) for triclinic box used in the SLLOD algorithm. (**a**) Triclinic box (red solid line) and its images (black dotted lines) due to PBCs; (**b**) Lees-Edwards PBCs, which are equivalent to the PBCs used with the triclinic box in (**a**).

To implement SLLOD, LAMMPS applies a triclinic box whose dimensions can be expressed as

(10) $$\left(a,b,c\right)=\left(\begin{array}{ccc} L_{x} & xy & xz\\ 0 & L_{y} & yz\\ 0 & 0 & L_{z} \end{array}\right),$$

where $L_{x}$, $L_{y}$, and $L_{z}$ are the lengths of the main cell of the triclinic box along the three Cartesian coordinates, and xy, yz, and xz are the tilt factors, which can assume positive, negative, or zero values. The triclinic box reduces to an orthogonal box when all tilt factors are 0. Tilt factors can be considered as displacements applied to the faces of an original orthogonal box in order to transform it into a parallelopiped, as shown in Figure 2a. In molecular simulations, the main cell of the triclinic box and its image cells produced via the use of periodic boundary conditions (PBCs) can be transformed into an equivalent setup shown in Figure 2b, which is called Lees-Edwards PBCs [49]. In the SLLOD method, a shear applied in the *x* direction and a velocity gradient applied in the *y* direction, increases the tilt factor xy gradually. When this tilt factor reaches $L_{x}/2$, it immediately changes to $-L_{x}/2$, and this switching pattern is repeated throughout the course of the simulation. Due to this specific use of the tilt factors and the associated complexity created by the PBCs, caution is needed in calculating the distances between particles in the main cell and those in the image cells in order to accurately compute the aforementioned orientation tensors.

### 2.4. Principal Component Analysis

Principal Component Analysis (PCA) [25] is a widely used dimension reduction method to visualize and analyze high-dimensional datasets. PCA implements a linear transformation of the variables in the original high-dimensional space to find new variables in the low-dimensional space. In order to preserve as much information as possible, the new variables are chosen such that the greatest variances are projected on these dimensions. To help understand this, we briefly explain the concepts of variance and covariance. Variance is the average squared deviation from the mean value for a particular variable (dimension):

(11) $$\mathrm{Var}\left(a\right)=\frac{1}{m}\sum_{i=1}^{m}{\left(a_{i}-\bar{a}\right)}^{2},$$

where *m* is the number of data points associated with that variable. Often, the data for each variable is shifted to render a mean value of 0 (i.e., $\bar{a}=0$), yielding

(12) $$\mathrm{Var}\left(a\right)=\frac{1}{m}\sum_{i=1}^{m}a_{i}^{2}=\frac{1}{m}\sum_{i=1}^{m}a_{i}a_{i}.$$

Variance represents the extent of separation in the data describing a particular variable.

Covariance between any two variables (e.g., *a* and *b*) represents the correlation between them and is calculated using the formula:

(13) $$\mathrm{Cov}\left(a,b\right)=\frac{1}{m}\sum_{i=1}^{m}a_{i}b_{i},$$

where we assume that $\bar{a}=\bar{b}=0$. While the variance is a strictly non-negative quantity, covariance can be positive or negative or zero depending on correlation of the two dimensions. A covariance of 0 indicates that the associated two variables are linearly independent, i.e., they represent orthogonal dimensions. In this sense, to reduce the data dimensions by PCA, one needs to find new variables with zero covariance (to keep them independent) and with the largest variances (to preserve as much information as possible).

Consider an n×m matrix *X* to represent an *n*—dimensional data where each dimension (variable) is represented with *m* data points:

(14) $$X=\left[\begin{array}{ccccc} x_{1}^{1} & x_{2}^{1} & x_{3}^{1} & \ldots & x_{m}^{1}\\ x_{1}^{2} & x_{2}^{2} & x_{3}^{2} & \ldots & x_{m}^{2}\\ \vdots & \vdots & \vdots & \ddots & \vdots \\ x_{1}^{n} & x_{2}^{n} & x_{3}^{n} & \ldots & x_{m}^{n} \end{array}\right].$$

Using *X*, an n×n matrix $C=\frac{1}{m}XX^{T}$ can be computed:

(15) $$C=\frac{1}{m}\sum_{i=1}^{m}\left[\begin{array}{ccccc} x_{i}^{1}x_{i}^{1} & x_{i}^{1}x_{i}^{2} & x_{i}^{1}x_{i}^{3} & \ldots & x_{i}^{1}x_{i}^{n}\\ x_{i}^{2}x_{i}^{1} & x_{i}^{2}x_{i}^{2} & x_{i}^{2}x_{i}^{3} & \ldots & x_{i}^{2}x_{i}^{n}\\ \vdots & \vdots & \vdots & \ddots & \vdots \\ x_{i}^{n}x_{i}^{1} & x_{i}^{n}x_{i}^{2} & x_{i}^{n}x_{i}^{3} & \ldots & x_{i}^{n}x_{i}^{n} \end{array}\right].$$

*C* can be seen as the variance-covariance matrix for the *n*-dimensional data, where the diagonal elements are the variances of the variables and the off-diagonal elements are the covariances between two different variables.

We seek a transformation of the original matrix *X* such that, in the space of the new variables, the variance-covariance matrix is a diagonal matrix, i.e., its off-diagonal elements (covariances) are 0. Let *P* (as yet unknown) be the transformation matrix operating on *X* that yields the matrix represented with the new, transformed variables Y=PX. The variance-covariance matrix for the transformed data is $D=\frac{1}{m}YY^{T}=\frac{1}{m}\left(PX\right){\left(PX\right)}^{T}=\frac{1}{m}PXX^{T}P^{T}=PCP^{T}$, where *C* is the matrix in Equation (15). In the light of our goal, *D* needs to be a diagonal matrix, which implies that we need to find *P* such that $PCP^{T}$ is a diagonal matrix. Given that $D=PCP^{T}$ is an n×n symmetric matrix, there always exists *n* orthonormal eigenvectors $E=(e_{1},e_{2},\ldots ,e_{n})$ that can diagonalize the matrix *C* [50]:

(16) $$ECE^{T}=\Lambda =\left[\begin{array}{cccc} \lambda_{1} & \; & & \\ \; & \lambda_{2} & \; & \\ \; & \; & \ddots & \\ \; & \; & & \lambda_{n} \end{array}\right],$$

where Λ is a diagonal matrix, whose diagonal elements are the eigenvalues corresponding to the eigenvectors. Equation (16) shows the desired matrix P=E. Every row of the n×n matrix *P* is an eigenvector of matrix *C*. If we sort the eigenvalues of Λ in a descending order, the result of the first *k* rows of the matrix *P* multiplying the original data *X* is the desired data in the reduced dimensions, and *k* becomes the number of reduced dimensions (principal components). The ratio of each eigenvalue to the summation of all eigenvalues approximately represents the weight fraction of the information in the original data that is captured by the corresponding reduced dimension (principal component).

### 2.5. t-Distributed Stochastic Neighbor Embedding

Unlike PCA, the stochastic neighbor embedding (SNE) [26] method is a non-linear technique for dimension reduction. The key idea underlying SNE is the transformation of the Euclidean distance between each pair of data points to a probability, which is used to represent pairwise similarity. A short distance indicates a high similarity, suggesting the associated data points have a high probability of belonging to the same cluster. In SNE, one calculates the pairwise similarity in both high-dimensional space and low-dimensional space, and then optimizes the overlap between these two similarities using a cost function.

In the high-dimensional space, a Gaussian distribution is centered around a data point $x_{i}$, and the probability of another data point $x_{j}$ to fall within this Gaussian distribution is measured:

(17) $$p_{j|i}=\frac{\exp\left(-\left|\right|x_{i}-x_{j}{\left|\right|}^{2}/\left(2\sigma_{i}^{2}\right)\right)}{\sum_{k\neq i}\exp\left(-\left|\right|x_{i}-x_{k}{\left|\right|}^{2}/\left(2\sigma_{i}^{2}\right)\right)},$$

where the probability is renormalized such that $\sum_{j}p_{j|i}=1$. $\sigma_{i}$ changes with data point $x_{i}$, and is related to a SNE tunable parameter, perplexity, which influences the number of nearest neighbors considered by SNE to obtain $p_{j|i}$. Suggested values of perplexity are between 5 and 50 [40]. After dimension reduction, the points in the high-dimensional space are mapped into the low-dimensional space ($x_{i}\to y_{i}$). A similar calculation is performed in the low-dimensional space to measure the probability of a point ($y_{j}$) to fall within the Gaussian distribution centered around a point $y_{i}$:

(18) $$q_{j|i}=\frac{\exp\left(-\left|\right|y_{i}-y_{j}{\left|\right|}^{2}/\left(2\sigma_{i}^{2}\right)\right)}{\sum_{k\neq i}\exp\left(-\left|\right|y_{i}-y_{k}{\left|\right|}^{2}/\left(2\sigma_{i}^{2}\right)\right)}.$$

The SNE transformation tries to fit the low-dimensional distribution to the high-dimensional distribution. The fit or “mutual consistency” of these two distributions can be measured by computing the cross entropy or Kullback-Leibler divergence (KLD) [51] defined in terms of the cost function:

(19) $$\mathrm{Cost}=\sum_{i}\sum_{j}p_{j|i}\log\frac{p_{j|i}}{q_{j|i}}.$$

The Cost function is asymmetric: it is positive when $p_{j|i}>q_{j|i}$, and negative when $p_{j|i}<q_{j|i}$. This has important implications: if the high-dimensional data are clustered, but they are separated in low dimensional space (i.e., $p_{j|i}>q_{j|i}$), the Cost function will apply a penalty. On the other hand, the Cost function will accept the scenario when $p_{j|i}<q_{j|i}$. Thus, SNE tries to preserve the local structure of the high-dimensional data.

The original SNE often suffers from the so-called “crowding problem”, which means that well separated data in the high-dimensional space are not well separated in the low-dimensional space. This problem can be mitigated by modifying the distribution function in the low-dimensional space to the “student’s t-distribution” (also known as “Cauchy distribution”). Its expression and associated $q_{j|i}$ [40] are given as:

(20) $$f\left(t\right)=\frac{1}{\pi (1+t^{2})};$$

(21) $$q_{j|i}=\frac{{\left(1+\left|\right|y_{i}-y_{j}{\left|\right|}^{2}\right)}^{-1}}{\sum_{k\neq i}{\left(1+\left|\right|y_{i}-y_{k}{\left|\right|}^{2}\right)}^{-1}}.$$

t-distributed SNE (t-SNE) is based on SNE with the distribution function replaced by the t-distribution in the low-dimensional space. t-distribution function has a heavier tail compared to Gaussian distribution, which enables a superior mapping for the well separated data points. In general, t-SNE can provide a good data visualization by capturing correlations not picked by linear methods such as PCA. However, t-SNE has a few drawbacks [52]. For example, the non-convex nature of the cost function can lead to variations in its minimum value during optimization. Furthermore, t-SNE is computationally expensive, scaling as $O(m^{2})$, where *m* is the number of data points.

## 3. Results and Discussion

We first summarize simulation results from our earlier work on the shear viscosity of squalane at different rates and pressures relevant to the studies performed in later subsections. Next, we present the examination of the link between rheological properties and molecular structure using the intramolecular orientation tensors. This is followed by results that probe this link using the intermolecular orientation tensors.

### 3.1. Rheological Properties

**Figure 3:**
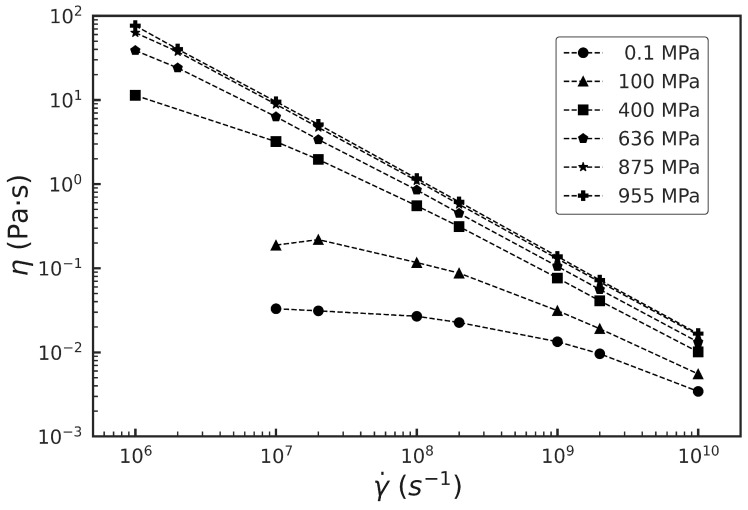
NEMD simulation results for the steady-state shear viscosity η of squalane at different strain rates $\dot{\gamma }$ for pressures shown in the legend. The results are at 293 K and obtained from Ref. [18].

Figure 3 shows the NEMD simulation results for the shear viscosity of squalane for different shear rates $\dot{\gamma }\in \left(10^{6}-10^{10}\right)$$s^{-1}$ and pressures P=0.1,100,400,636,875,955 MPa at 293 K. Shear viscosity η is calculated as $\eta =-\sigma_{xy}/\dot{\gamma }$, where $\sigma_{xy}$ is the xy component of the stress tensor. At all pressures, squalane exhibits a clear shear thinning behavior evident by a decreasing viscosity with increasing shear strain rate. Our previous papers [17,18] showed that phenomenological models and the associated molecular mechanisms that best describe the observed shear thinning behavior can be different at different pressures. For low pressures P≤ 100 MPa, where the Newtonian viscosity $\eta_{N}$ is small, the viscosity of squalane is better fit by power-law models such as Carreau model and Ostwald-de Waele model [53,54], which predict a power-law shear thinning as a function of rate: $\eta =\eta_{N}{\left(1+{\left(\dot{\gamma }/{\dot{\gamma }}_{0}\right)}^{2}\right)}^{\frac{n-1}{2}}$, where ${\dot{\gamma }}_{0}$ is a characteristic shear rate, and the exponent *n* is between 0 and 1. For low rates $\dot{\gamma }\ll {\dot{\gamma }}_{0}$, η reduces to the Newtonian viscosity $\eta_{N}$. For rates $\dot{\gamma }\gg {\dot{\gamma }}_{0}$, η shows power-law behavior. Our earlier work showed that the viscosity extracted from the Newtonian plateau reached in simulations and via the fit to the Carreau model is in excellent agreement with the Newtonian viscosity measured in experiments [17,18]. In such models, shear thinning is attributed to changes in molecular order. For example, an increase in the molecular alignment along the flow direction with increasing rate can reduce the rate of collisions between molecules, and thus decrease the fluid viscosity.

On the other hand, for high pressures P≥ 400 MPa, where $\eta_{N}$ is large, the viscosity-rate scaling is better fit by Eyring model [55,56], which predicts a logarithmic rise in shear stress with rate, resulting in the limiting high-rate shear thinning behavior: $\eta =\left(\eta_{N}{\dot{\gamma }}_{E}/\dot{\gamma }\right)\log\left(2\dot{\gamma }/{\dot{\gamma }}_{E}\right)$ for $\dot{\gamma }\gg {\dot{\gamma }}_{E}$, where ${\dot{\gamma }}_{E}$ is a characteristic rate related to the Eyring stress parameter $\sigma_{E}=\eta_{N}{\dot{\gamma }}_{E}$. Our previous papers showed that Eyring model fits to high-rate viscosity data from simulations were consistent with experimental measurements of viscosity at the same pressure that reach up to $10^{4}$ $s^{-1}$ and extend down into the Newtonian regime [17,18]. Eyring model assumes that shear flow is a stress-biased thermally activated process, where shear occurs through rare molecular rearrangements that require activation over a single potential energy barrier. In Eyring model, the reduction in viscosity with shear rate does not rely on a change of molecular order, but instead on a change of the competition between shear rate and thermal activation.

Given these alternate molecular mechanisms of shear thinning, one that relies on changes in molecular order and the other that does not, an accurate assessment of changes in molecular order with increasing shear rate for different pressures becomes important to probe the origins of the observed high-rate shear thinning behavior shown in Figure 3. In the following, we examine the link between shear thinning and molecular order in squalane for different pressures by using the molecular structure information encoded in the intramolecular and intermolecular orientation tensors.

### 3.2. Intramolecular Orientation and Shear Thinning: Single Atom Pair Analysis

The intramolecular orientation tensor $S_{\alpha \beta }^{p}$ (Equation (7)) is defined for each (united-) atom pair *p* associated with a squalane molecule. A squalane molecule has 30 united atoms, and therefore there are 435 intramolecular atom pairs. The intramolecular structure information is encoded in the associated 435 intramolecular orientation tensors. Each of these $S_{\alpha \beta }^{p}$ tensors is a 3 × 3 symmetric matrix, where $S_{\alpha \beta }^{p}$ = $S_{\beta \alpha }^{p}$. Therefore, 6 out of 9 components are non-trivial. The diagonal elements $S_{\alpha \alpha }^{p}$ measure the degree of atom pair alignment in the direction of the flow field (*x*), velocity gradient (*y*), and vorticity (*z*), respectively. A value of 0 indicates no preference to align along the axis, i.e., the atom-pair distance vector assumes a random orientation. Positive and negative values imply the extent to which the atom-pair orientation is parallel or perpendicular to the axis, with 1 and −0.5 representing perfectly parallel and perpendicular alignments, respectively.

**Figure 4:**
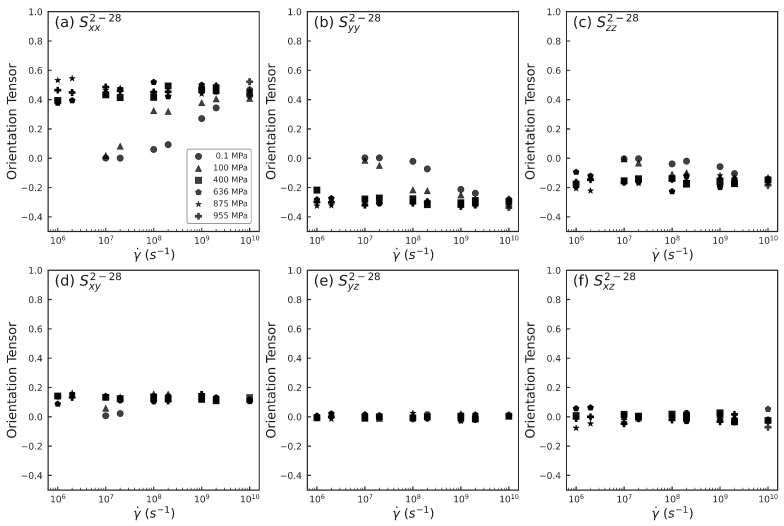
Intramolecular orientation tensor components: (**a**) $S_{xx}^{2-28}$, (**b**) $S_{yy}^{2-28}$, (**c**) $S_{zz}^{2-28}$, (**d**) $S_{xy}^{2-28}$, (**e**) $S_{yz}^{2-28}$, and (**f**) $S_{xz}^{2-28}$ associated with the atom pair 2−28 (Figure 1) vs. strain rate $\dot{\gamma }$ for pressures listed in (**a**). *x*, *y*, and *z* represent the flow, velocity gradient, and vorticity directions respectively.

To illustrate, Figure 4 shows the intramolecular orientation tensor for the atom pair 2−28 (see Figure 1) for different rates and pressures. For pressures P≤ 100 MPa, $S_{xx}^{2-28}$, $S_{yy}^{2-28}$, and $S_{zz}^{2-28}$ (Figure 4a–c) start at ≈0 at the lowest strain rates probed ($\dot{\gamma }=10^{7}$$s^{-1}$) and gradually increase or decrease with increasing rate, before plateauing to a non-zero value at the highest rates ($\dot{\gamma }=10^{10}$$s^{-1}$). This behavior signals a gradual shift in the orientation of the atom pair 2−28 from a random orientation to an ordered orientation as shear rate increases from $10^{7}$$s^{-1}$ to $10^{10}$$s^{-1}$. The high-rate plateau values for $S_{xx}^{2-28}$, $S_{yy}^{2-28}$, and $S_{zz}^{2-28}$ are positive, negative, and negative, respectively, which indicates that the atom pair 2−28 orients along the flow direction while being perpendicular to the velocity gradient and vorticity directions.

In stark contrast, the diagonal components $S_{xx}^{2-28}$, $S_{yy}^{2-28}$, and $S_{zz}^{2-28}$ for pressures P≥ 400 MPa start at non-zero values at the lowest probed rates ($\dot{\gamma }=10^{6}$$s^{-1}$) and do not change noticeably as $\dot{\gamma }$ increases to $10^{10}$$s^{-1}$. This behavior signals that the atom pair 2−28 does not have an appreciable change in the orientation even as squalane is sheared at increasingly higher rates. Figure 4d shows the $S_{xy}^{2-28}$ component for all the pressures. A trend similar to $S_{xx}^{2-28}$ is observed: $S_{xy}^{2-28}$ increases from ≈0 to a saturated positive value with increasing $\dot{\gamma }$ for P≤ 100 MPa, but is nearly constant at a positive saturated value for P≥ 400 MPa. For all pressures, the off-diagonal components $S_{yz}^{2-28}$ and $S_{xz}^{2-28}$ exhibit small fluctuations around zero for all rates.

For higher pressures, even though all the orientation tensor components associated with the 2−28 atom pair do not change significantly with increasing rate, the viscosity continues to steadily decrease. This shear thinning may result from either a change in a different intramolecular order parameter (e.g., orientation of a different atom pair), or from an entirely different molecular mechanism (e.g., thermal activation). Different atom pairs might contribute differently to the molecular order, and therefore the orientation tensor information associated with not just 1, but all 435 pairs needs to be examined. We next show the results of using dimension reduction methods to analyze and visualize this high-dimensional information.

### 3.3. Intramolecular Orientation and Shear Thinning: Dimension Reduction Results

We first classify the 30 united atoms of a squalane molecule into 3 types based on their positions (see Figure 1): end atoms (atoms 1, 3, 29, and 30), side atoms (atoms 8, 13, 19, and 24), and backbone atoms (all the other atoms in Figure 1). Using this classification, we separate the 435 atom pairs into 4 representative pair types: side-backbone pairs of a side atom and its bonded backbone atom (e.g., atom pair 7–8 in Figure 1), end-backbone pairs of an end atom and its bonded backbone atom (e.g., atom pair 2–1 in Figure 1), short backbone-backbone pairs of two backbone atoms directly connected by a covalent bond (e.g., atom pair 15–16 in Figure 1), and long backbone-backbone pairs of two backbone atoms that are separated by more than one covalent bond (e.g., atom pair 2–28 in Figure 1 which is separated by 21 bonds).

Each of the 435 atom pairs is associated with an intramolecular orientation tensor having 6 non-trivial components. This high-dimensional dataset of 6×435 dimensions encapsulates the intramolecular structure information for each pressure and shear rate. In the following, we discuss the results of using PCA and t-SNE to reduce and visualize this high-dimensional information. In all dimension reduction tasks, each component of the orientation tensors is first centralized and normalized such that the mean and variance of the data points are 0 and 1 respectively.

**Figure 5:**
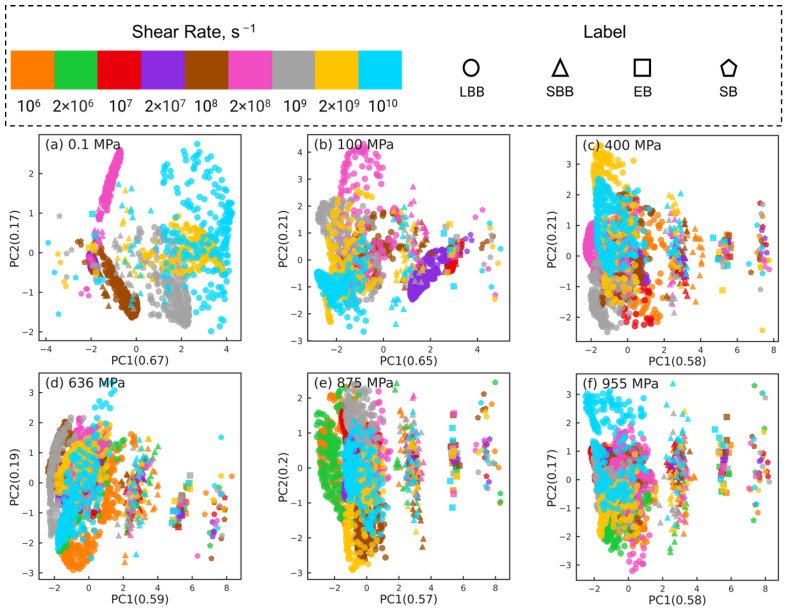
Dimension reduction using PCA of intramolecular orientation tensors associated with all atom pairs of squalane sheared at six different pressures: (**a**) 0.1 MPa, (**b**) 100 MPa, (**c**) 400 MPa, (**d**) 636 MPa, (**e**) 875 MPa, and (**f**) 955 MPa. Each point represents one pair orientation tensor. For clarity, tensors corresponding to the 4 representative atom pair types (described in the text) are shown: side-backbone (SB), end-backbone (EB), short backbone-backbone (SBB), and long backbone-backbone (LBB). Different colors denote different shear rates and different symbols represent different atom pair types, as shown in the legend panel at the top.

Figure 5 shows the PCA results for reducing the datasets of $n_{r}\times 6\times 435$ dimensions to $n_{r}\times 2\times 435$ dimensions at different pressures, where $n_{r}$ indicates the number of shear rates associated with the corresponding pressure ($n_{r}=7$ for 0.1 and 100 MPa, $n_{r}=8$ for 400 MPa, and $n_{r}=9$ for P≥636 MPa). For clarity, orientation tensors associated with the aforementioned 4 pair types (i.e., side-backbone, end-backbone, short backbone-backbone, long backbone-backbone pairs) are shown in Figure 5. The sub-figures correspond to different pressures P= 0.1 MPa (a), 100 MPa (b), 400 MPa (c), 636 MPa (d), 875 MPa (e), and 955 MPa (f). In each sub-figure, the atom pairs are identified by the shear rate (symbol color) and the pair type: side-backbone, end-backbone, short backbone-backbone, long backbone-backbone (symbol type), as illustrated in the legend. The first and second principal components (PC1 and PC2) represent the components associated with the reduced dimensional space that, respectively, capture the largest and the second largest variance in the high-dimensional dataset. The numbers in the parentheses of PC1 and PC2 indicate the weight fraction of the information in the original data that is captured by the corresponding component. For example, in Figure 5a, PC1 and PC2 capture 67% and 17% of the information embedded in the high-dimensional space, respectively. All the other components combined capture the remaining 16% of the information in the original data.

For all 6 pressures, by analyzing the eigenvectors associated with PC1 and PC2, we find that $S_{xx}$, $S_{yy}$, $S_{zz}$, and $S_{xy}$ components contribute significantly to PC1, while PC2 is dominated by $S_{yz}$ and $S_{xz}$ components. This suggests that dimension reduction using PCA picks a low-dimensional axis that represents the shear-induced changes in the intramolecular orientation tensors as the first principal component. The $S_{yz}$ and $S_{xz}$ components that dominate PC2 are expected to be 0 on average, which suggests that the second most important dimension in the low-dimensional space represents the variations in the intramolecular orientation tensors that can be attributed to statistical fluctuations.

The PCA dimension reduction results for pressures P=0.1,100 MPa (Figure 5a,b) show that the atom pairs are better separated by strain rate (symbol color) compared to by pair type (symbol type). The datasets associated with the lowest rates, where squalane exhibits Newtonian flow, are not separable, consistent with our earlier work [24]. Most atom pairs associated with the same shear rate are grouped together into clusters that are organized along the PC1 dimension by the strain rate, e.g., from left to right with increasing rate for 0.1 MPa. In stark contrast, PCA dimension reduction results for pressures P=400,636,875,955 MPa (Figure 5c–f) show that the atom pairs are better separated by pair type (symbol type) compared to by strain rate (symbol color). The side-backbone, end-backbone, short backbone-backbone, and long backbone-backbone pairs organize into distinct clusters well-separated along the PC1 axis. The large separation between clusters comprising side-backbone and long backbone-backbone pairs suggests that these pairs contribute to the variations in the intramolecular orientation tensors in contrasting roles.

**Figure 6:**
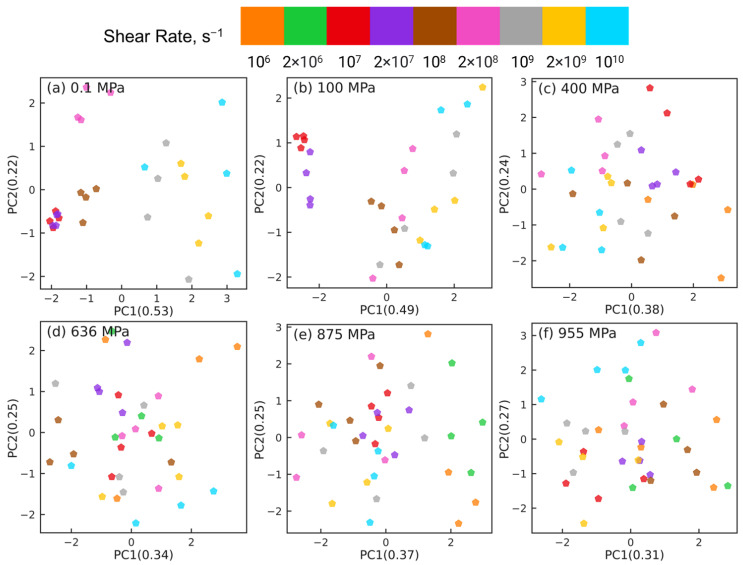
Dimension reduction using PCA of intramolecular orientation tensors associated with the side-backbone atom pairs of squalane sheared at pressures: (**a**) 0.1 MPa, (**b**) 100 MPa, (**c**) 400 MPa, (**d**) 636 MPa, (**e**) 875 MPa, and (**f**) 955 MPa. Each point represents one pair orientation tensor. Different colors denote different shear rates as shown in the legend panel at the top.

**Figure 7:**
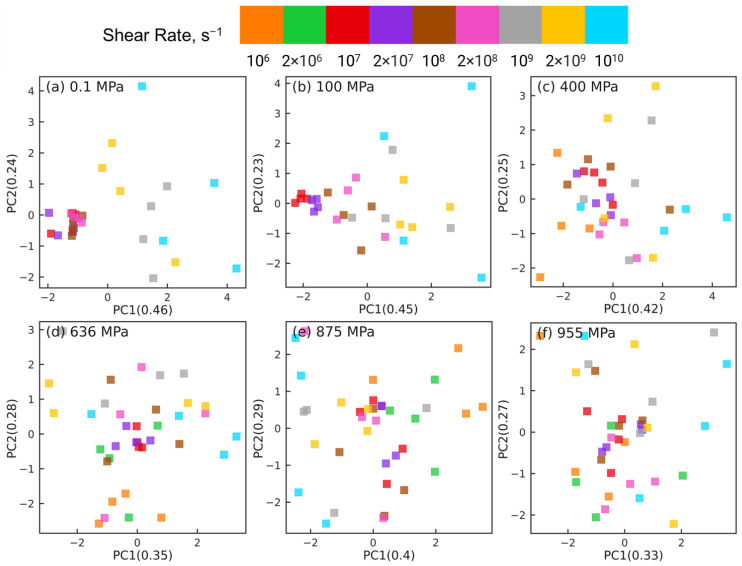
Dimension reduction using PCA of intramolecular orientation tensors associated with the end-backbone atom pairs of squalane sheared at pressures: (**a**) 0.1 MPa, (**b**) 100 MPa, (**c**) 400 MPa, (**d**) 636 MPa, (**e**) 875 MPa, and (**f**) 955 MPa. Each point represents one pair orientation tensor. Different colors denote different shear rates as shown in the legend panel at the top.

**Figure 8:**
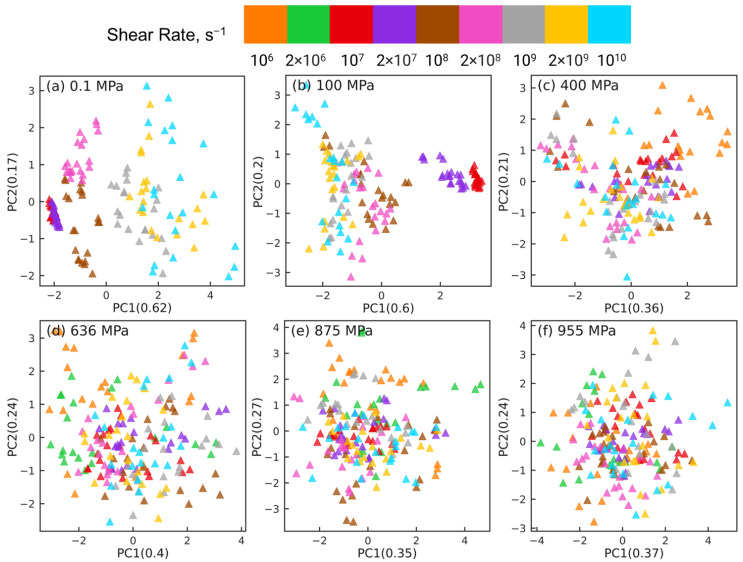
Dimension reduction using PCA of intramolecular orientation tensors associated with the short backbone-backbone atom pairs of squalane sheared at pressures: (**a**) 0.1 MPa, (**b**) 100 MPa, (**c**) 400 MPa, (**d**) 636 MPa, (**e**) 875 MPa, and (**f**) 955 MPa. Each point represents one pair orientation tensor. Different colors denote different shear rates as shown in the legend panel at the top.

**Figure 9:**
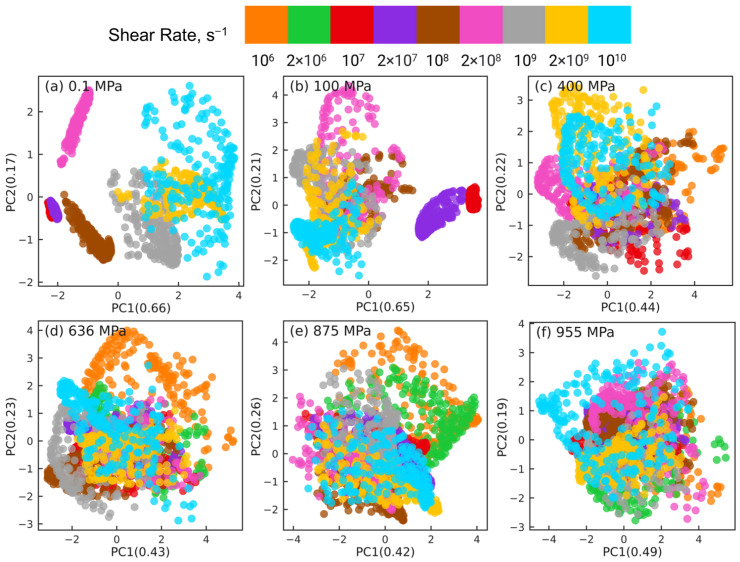
Dimension reduction using PCA of intramolecular orientation tensors associated with the long backbone-backbone atom pairs of squalane sheared at pressures: (**a**) 0.1 MPa, (**b**) 100 MPa, (**c**) 400 MPa, (**d**) 636 MPa, (**e**) 875 MPa, and (**f**) 955 MPa. Each point represents one pair orientation tensor. Different colors denote different shear rates as shown in the legend panel at the top.

The PCA results shown in Figure 5 highlight that the changes in the intramolecular orientation tensors of all 435 atom pairs with increasing shear rate at low pressures (≤100 MPa) are dramatically different from the changes that occur when squalane is sheared over similar range of strain rates at high pressures. It is informative to examine if these differences persist when the intramolecular orientation tensors associated with atom pairs of only one specific type are examined using PCA. Figure 6, Figure 7, Figure 8 and Figure 9 show the PCA results for reducing the dimension of intramolecular orientation tensor datasets for side-backbone pairs, end-backbone pairs, short backbone-backbone pairs, and long backbone-backbone pairs, respectively, at different pressures. At low pressures P≤ 100 MPa, regardless of the pair type, the atom pairs are organized along the PC1 axis by strain rate. This indicates that rate-dependent shear-induced changes in the intramolecular orientation tensors dominate the overall intramolecular structure evolution at low pressures, and thus can be linked to the reduction in the viscosity of squalane. At high pressures P≥ 400 MPa, the side-backbone, end-backbone, short backbone-backbone, and long backbone-backbone atom pairs are dispersed and they do not organize in any systematic way. This signals an overall limited evolution of molecular orientation of these atom pairs with changes in shear rate. As a result, the shear thinning of squalane observed for these pressures can not be explained via the evolution in the intramolecular orientation tensors associated with atom pairs of these 4 pair types.

**Figure 10:**
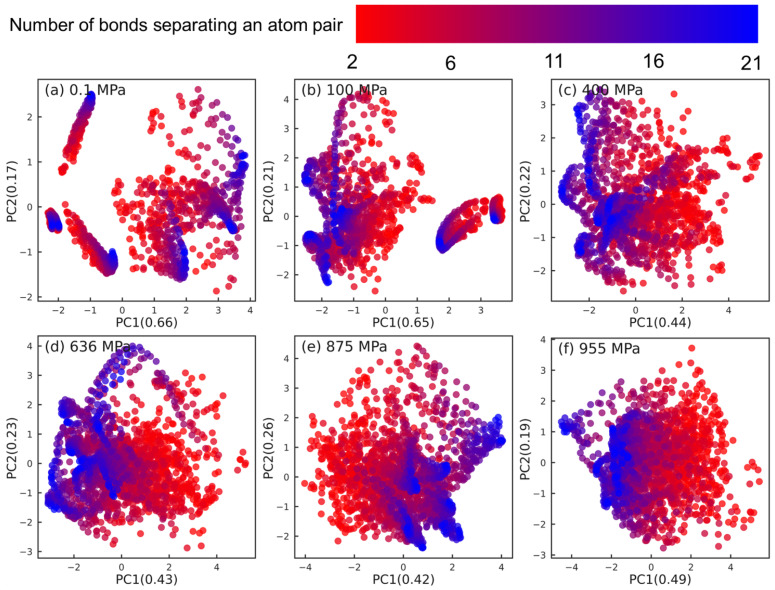
Dimension reduction using PCA of intramolecular orientation tensors associated with the long backbone-backbone atom pairs of squalane sheared at pressures: (**a**) 0.1 MPa, (**b**) 100 MPa, (**c**) 400 MPa, (**d**) 636 MPa, (**e**) 875 MPa, and (**f**) 955 MPa. Each point represents one pair orientation tensor. Different colors denote different number of bonds separating an atom pair, as shown in the top legend. Atom-pair separation increases as symbol color changes from red to blue.

While the long backbone-backbone atom pairs associated with different strain rates are mixed together in Figure 9, the overall large number of these pairs and the wide distribution of separation bonds (from 2 to 21) associated with these pairs might blur the information in the low-dimensional space. Therefore, we divide this group into 20 sub-groups where each group is characterized and distinguished by an integer bond separation number b∈[2,21] representing the number of bonds separating an atom pair. Figure 10 shows the same PCA dimension reduction results shown in Figure 9, except, instead of strain rate, the symbols are colored with the number of bonds separating the atom pair (i.e., the bond separation number). A red-blue color gradient scale is used where red represents smaller bond separation numbers (e.g., b=2,6) and blue represents higher bond separation numbers (e.g., b=16,21). At low pressures P=0.1,100 MPa, the red and blue symbols appear mixed with no clear organization along either principal components. Interestingly, unlike Figure 9c–f, a pattern in the organization of atom pairs for higher pressures P=400,636,875,855 MPa emerges in Figure 10c–f: the atom pairs are organized along the PC1 axis in increasing or decreasing order by their bond separation number. In each case, the far-separated atom pairs with large *b* values (blue symbols) are clearly separated from the near-separated atom pairs characterized with small *b* values (red symbols).

**Figure 11:**
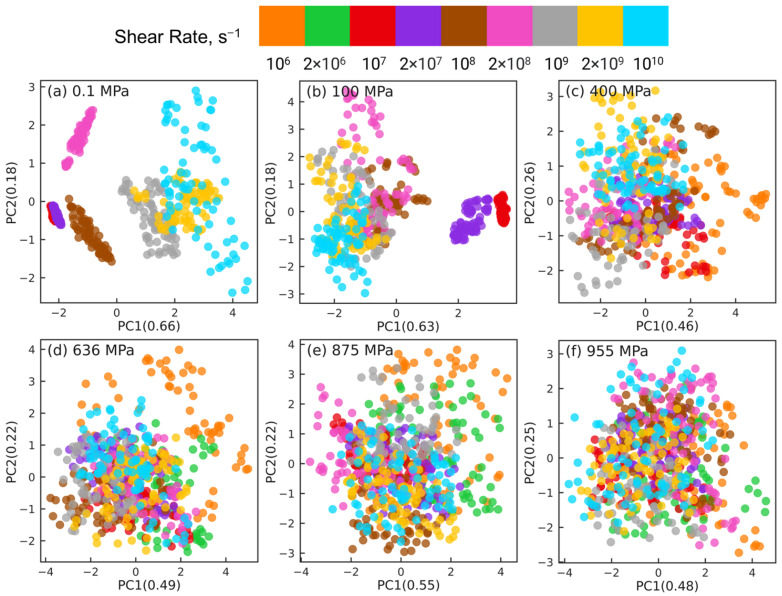
Dimension reduction using PCA of intramolecular orientation tensors associated with the near pairs of squalane sheared at pressures: (**a**) 0.1 MPa, (**b**) 100 MPa, (**c**) 400 MPa, (**d**) 636 MPa, (**e**) 875 MPa, and (**f**) 955 MPa. A “near pair” is defined as a long backbone-backbone pair separated by fewer than 6 bonds. Each point represents one pair orientation tensor. Different colors denote different shear rates as shown in the legend panel at the top.

The emergence of organization of atom pairs at higher pressures in the reduced dimensions motivates a further classification of the long backbone-backbone pairs into near pairs, which are atom pairs separated by more than 1 but fewer than 6 bonds (i.e., 1 <b<6), and far pairs, which are atom pairs separated by more than 16 but fewer than 22 bonds (16<b<22). We now repeat the dimension reduction tasks using PCA for these near and far long backbone-backbone pairs. Figure 11 shows the PCA results for near pairs at different pressures. At low pressures P≤ 100 MPa, atom pairs associated with the same strain rate are grouped together into clusters that are organized along the PC1 axis by strain rate. However, the atom pairs associated with different rates mix together and are not separable at high pressures P≥ 400 MPa. Thus, similar to the side-backbone, end-backbone, and short backbone-backbone atom pairs, the near long backbone-backbone pairs are also unable to link the observed reduction in viscosity of squalane with increasing rate to the evolution of the associated intramolecular orientation tensors.

**Figure 12:**
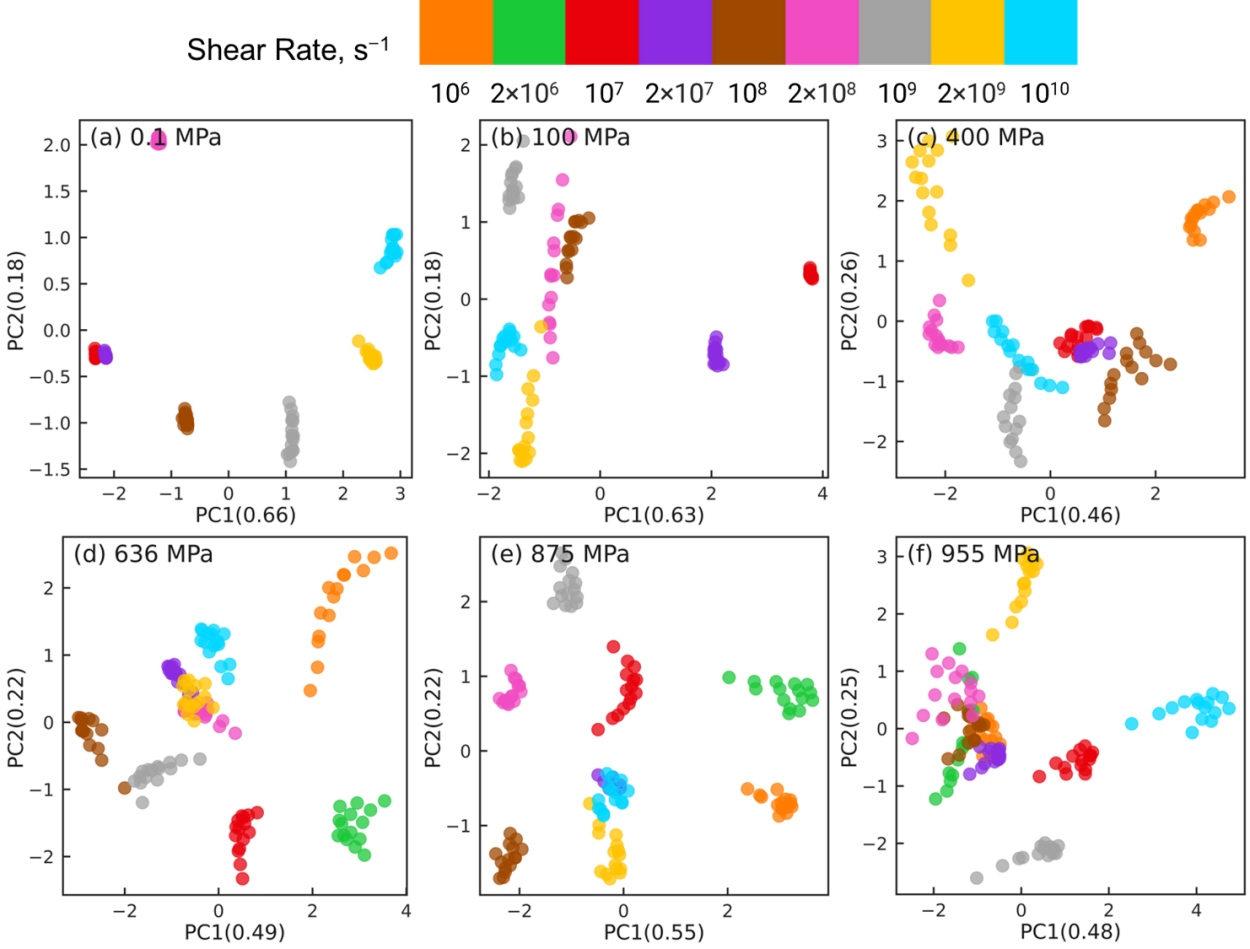
Dimension reduction using PCA of intramolecular orientation tensors associated with the far pairs of squalane sheared at pressures: (**a**) 0.1 MPa, (**b**) 100 MPa, (**c**) 400 MPa, (**d**) 636 MPa, (**e**) 875 MPa, and (**f**) 955 MPa. A “far pair” is defined as a long backbone-backbone pair separated by more than 16 bonds. Each point represents one pair orientation tensor. Different colors denote different shear rates as shown in the legend panel at the top.

Figure 12 shows the PCA results for far pairs at different pressures. A distinct picture emerges: for all pressures, the far atom pairs are grouped together into clusters that are separable by shear rates. At low pressures (P≤ 100 MPa), the separation between these clusters is more significant, and the clusters are organized along the PC1 axis by strain rate, similar to all other pair types (e.g., side-backbone, end-backbone, short backbone-backbone, and the near long backbone-backbone pairs). On the other hand, Figure 12c–f show that at high pressures P≥ 400 MPa, while the clusters of atom pairs associated with the same rate are still separable, their distribution in the low-dimensional space is random and not organized along either of the principal components. In other words, the clusters of atom pairs identified by the strain rate do not show a correlation with changes in the rate, as observed for low pressures in Figure 12a,b, where atom pairs are organized along PC1 axis by increasing or decreasing shear rate.

We note that well-separated clusters do not necessarily imply a significant evolution in the intramolecular structure with rate. From the single-pair results shown in Figure 4, we find that all the tensor components at high pressures are saturated and exhibit very small fluctuations with changes in strain rate. Because we normalize the data during the preprocessing stage, small fluctuations in these saturated values can get magnified and picked up by PCA during the dimension reduction. The enhanced separation between clusters of atom pairs can thus emerge from the differences between the magnified fluctuations associated with different rates. Therefore, in addition to the separation between clusters, it is also important to look at their organization in the reduced dimensions, which is random in Figure 12 suggesting the lack of correlation between shear thinning and changes in molecular orientation with strain rate.

**Figure 13:**
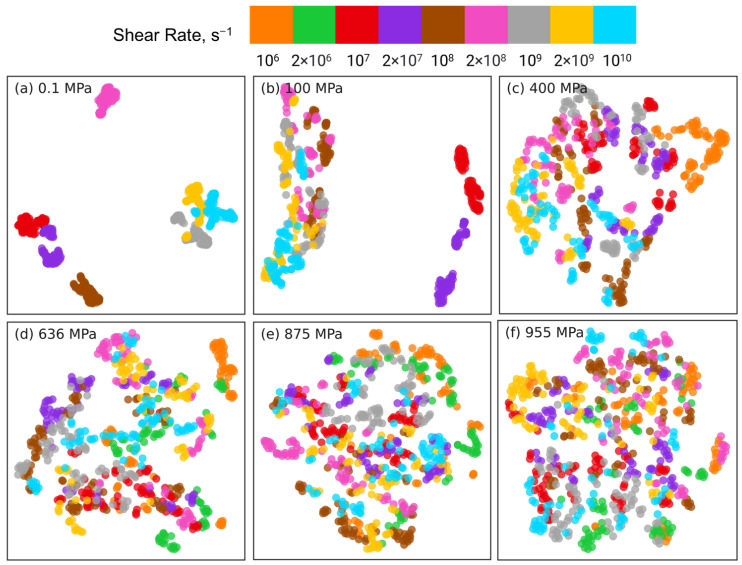
Dimension reduction using t-SNE of intramolecular orientation tensors associated with the near pairs of squalane sheared at pressures: (**a**) 0.1 MPa, (**b**) 100 MPa, (**c**) 400 MPa, (**d**) 636 MPa, (**e**) 875 MPa, and (**f**) 955 MPa. A “near pair” is defined as a long backbone-backbone pair separated by fewer than 6 bonds. Each point represents one pair orientation tensor. Different colors denote different shear rates as shown in the legend panel at the top.

**Figure 14:**
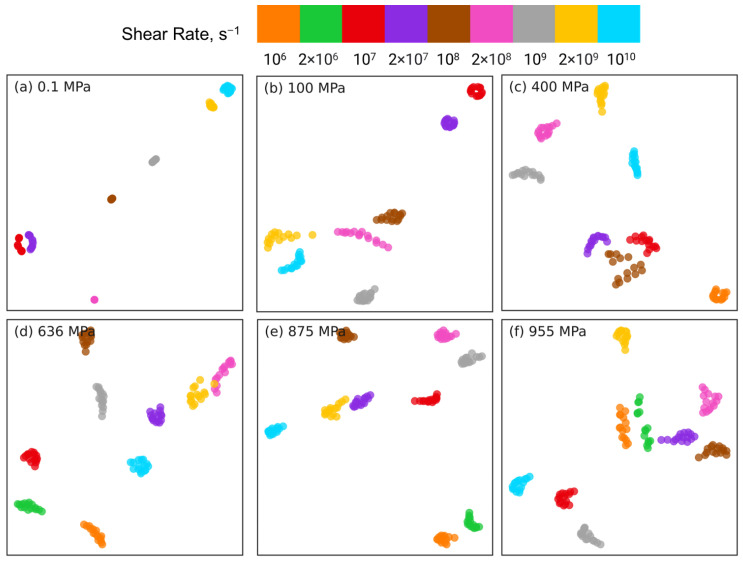
Dimension reduction using t-SNE of intramolecular orientation tensors associated with the far pairs of squalane sheared at pressures: (**a**) 0.1 MPa, (**b**) 100 MPa, (**c**) 400 MPa, (**d**) 636 MPa, (**e**) 875 MPa, and (**f**) 955 MPa. A “far pair” is defined as a long backbone-backbone pair separated by more than 16 bonds. Each point represents one pair orientation tensor. Different colors denote different shear rates as shown in the legend panel at the top.

To cross-check the PCA dimension reduction results for near and far long backbone-backbone pairs, we use t-SNE to reduce the dimension of intramolecular orientation tensor datasets associated with these 2 types of pairs. Figure 13 and Figure 14 show the t-SNE dimension reduction results for near pairs and far pairs, respectively, at different pressures. For near pairs, the data points with different shear rates are separable at low pressures *P* = 0.1, 100 MPa in Figure 13a,b, the separation is better for 0.1 MPa compared to 100 MPa. The clusters of atom pairs are also organized with shear rate along the horizontal direction, similar to the PCA results in Figure 11a,b. On the other hand, at high pressures P≥ 400 MPa in Figure 13c–f, the atom pairs are not separable and are scattered all over the plot. Figure 14a,b shows that at low pressures *P* = 0.1, 100 MPa, the far long backbone-backbone atom pairs with different shear rates are clearly separated and well organized with increasing or decreasing shear rate along the diagonal of the plot (e.g., from bottom left to top right for P=0.1 MPa). In contrast, while the far atom pairs associated with the same shear rate are grouped together into well-separated clusters for high pressures P≥ 400 MPa in Figure 14c–f, these clusters are randomly distributed, and do not exhibit any organization as observed in Figure 14a,b. Overall, t-SNE corroborates many of the PCA findings.

### 3.4. Intermolecular Orientation and Shear Thinning

The analysis based on the dimension reduction of the orientation tensors associated with intramolecular atom pairs does not consider effects arising from the interactions between the molecules on the viscosity of squalane. These intermolecular effects, such as the friction between a pair of molecules, can change with strain rate and pressure, thus altering the squalane viscosity. We now examine the relationship between intermolecular orientation and shear thinning. An intermolecular atom pair is defined for atoms belonging to two different squalane molecules. This atom pair is characterized with a separation distance parameter *d* which is chosen between 3.9 Å (an approximate value of the united-atom diameter) and 10 Å (the LJ potential cut-off distance). Discretizing this distance interval with a step size of 0.1 Å, 61 intermolecular atom pairs are generated for each strain rate and pressure. An intermolecular orientation tensor $S_{\alpha \beta }^{p}$ is computed for each pair *p* belonging to the set of these 61 pairs using Equation (7), where the ensemble average is taken over the number of atom pairs belonging to different squalane molecules that are associated with a given distance *d*. These 61 orientation tensors encapsulate the intermolecular orientation information at each strain rate and pressure.

**Figure 15:**
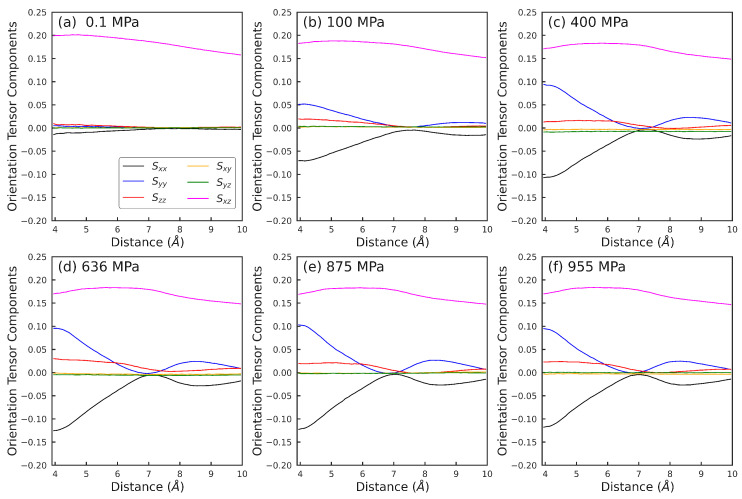
Intermolecular orientation tensor components at $\dot{\gamma }=10^{8}$$s^{-1}$ vs. distance between the atoms belonging to two different squalane molecules at pressures: (**a**) 0.1 MPa, (**b**) 100 MPa, (**c**) 400 MPa, (**d**) 636 MPa, (**e**) 875 MPa, and (**f**) 955 MPa. At each pressure, 6 lines correspond to the 6 components, where each line is represented with 61 points corresponding to the 61 atom pairs encapsulating the intermolecular structure information. *x*, *y*, and *z* represent the flow, velocity gradient, and vorticity directions respectively.

To illustrate, Figure 15 shows the 61 intermolecular orientation tensors at a strain rate of $\dot{\gamma }=10^{8}$$s^{-1}$ for pressures P=0.1,100,400,636,875,955 MPa. The diagonal elements $S_{xx}$, $S_{yy}$, and $S_{zz}$ of the orientation tensor at a distance d≈ 4 Å increasingly deviate from zero as pressure *P* increases from 0.1 MPa to 100 MPa, before plateauing for P≥400 MPa. As the distance *d* between the intermolecular atom pair increases, the diagonal elements of the corresponding orientation tensors exhibit a non-monotonic trend. For example, $S_{xx}$ exhibits an oscillatory behavior with increasing *d*, reaching a maximum value of 0 at d≈ 7−8 Å, and a minimum at ≈8−9 Å. $S_{yy}$ is nearly symmetric to $S_{xx}$ around the axis of zero orientation. $S_{zz}$ also oscillates with a relatively smaller amplitude and has a minimum near d≈ 7−8 Å. The off-diagonal elements do not change significantly with either pressure or the pair distance. $S_{xz}$ exhibits mild variations within the range of ≈0.15−0.2, and $S_{xy}$ and $S_{yz}$ are ≈0 for all *d* and *P*.

**Figure 16:**
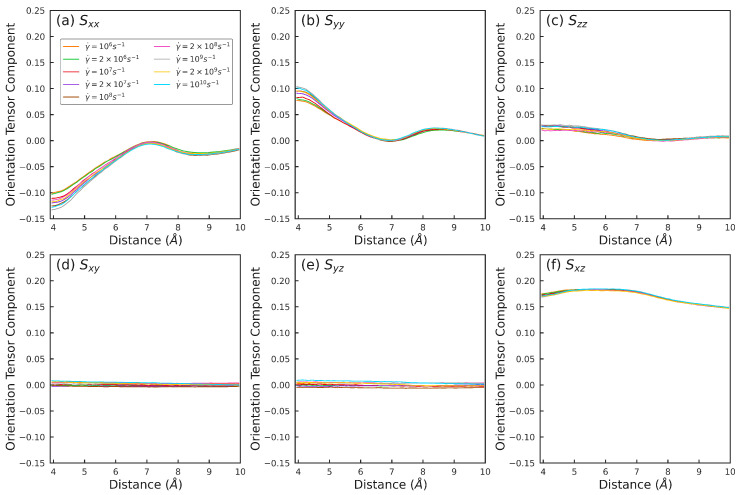
Intermolecular orientation tensor components: (**a**) $S_{xx}$, (**b**) $S_{yy}$, (**c**) $S_{zz}$, (**d**) $S_{xy}$, (**e**) $S_{yz}$, and (**f**) $S_{xz}$ vs. distance between the atoms belonging to two different squalane molecules at 636 MPa and strain rates $\dot{\gamma }$ noted in the legend in (**a**). Within each sub-figure, each line is represented with 61 points corresponding to the 61 atom pairs encapsulating the intermolecular structure information. *x*, *y*, and *z* represent the flow, velocity gradient, and vorticity directions respectively.

Figure 16 shows the 61 intermolecular orientation tensors at a high pressure P=636 MPa for different strain rates $\dot{\gamma }\in \left(10^{6}–10^{10}\right)$$s^{-1}$. The 6 sub-figures show the 6 tensor components: $S_{xx}$, $S_{yy}$, $S_{zz}$, $S_{xy}$, $S_{yz}$, and $S_{xz}$. Figure 16a–c show the variation of the diagonal elements $S_{xx}$, $S_{yy}$, and $S_{zz}$ as the separation distance *d* between the atoms is increased. For small d≈4–5Å, these components are different for different rates and assume non-zero values; the deviation from 0 is larger for higher strain rates. As *d* increases, $S_{xx}$, $S_{yy}$, and $S_{zz}$ approach 0 in a non-monotonic manner. Variations in these components with rate diminishes with increasing *d* such that for d≳7Å, $S_{xx}$, $S_{yy}$, and $S_{zz}$ are nearly the same for all rates. The off-diagonal components $S_{xy}$, $S_{yz}$, and $S_{xz}$ do not change significantly with either rate or *d*.

**Figure 17:**
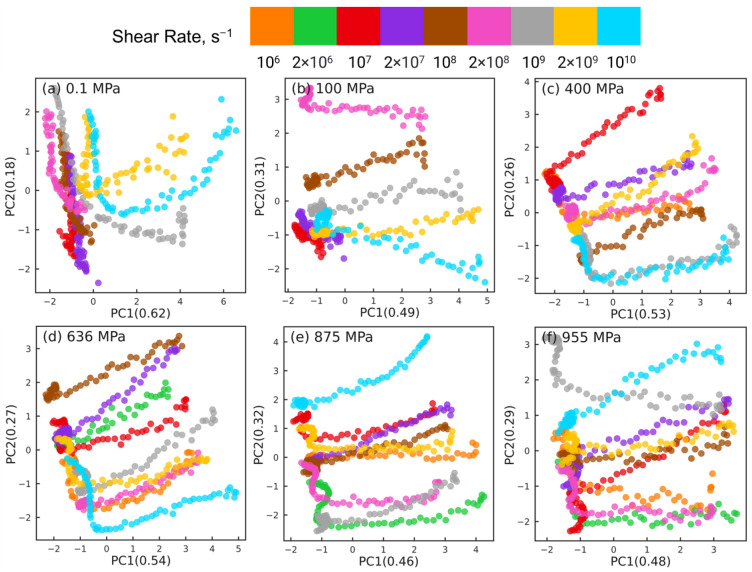
Dimension reduction using PCA of 61 intermolecular orientation tensors describing the intermolecular structure of squalane sheared at six different pressures: (**a**) 0.1 MPa, (**b**) 100 MPa, (**c**) 400 MPa, (**d**) 636 MPa, (**e**) 875 MPa, and (**f**) 955 MPa. Each point represents one pair orientation tensor. Different colors denote different shear rates as shown in the legend panel at the top.

Figure 17 shows the results for reducing the intermolecular orientation tensor datasets of $n_{r}\times 6\times 61$ dimensions to $n_{r}\times 2\times 61$ dimensions using PCA at different pressures, where $n_{r}$ indicates the number of shear rates associated with the corresponding pressure ($n_{r}=7$ for 0.1 and 100 MPa, $n_{r}=8$ for 400 MPa, and $n_{r}=9$ for P≥636 MPa.) The sub-figures correspond to different pressures P= 0.1 MPa (a), 100 MPa (b), 400 MPa (c), 636 MPa (d), 875 MPa (e), and 955 MPa (f). In each sub-figure, different colors are used to label the atom pairs associated with different shear rates. For all 6 pressures, by analyzing the eigenvectors associated with PC1 and PC2, we find that the non-zero components: $S_{xx}$, $S_{yy}$, $S_{zz}$, and $S_{xz}$ contribute significantly to PC1, while PC2 is dominated by $S_{xy}$ and $S_{yz}$ components, which are nearly zero.

Figure 17a,b show the dimension reduction results at low pressures P=0.1,100 MPa. These plots look distinct from Figure 17c–f which show the dimension reduction results at higher pressures P=400,636,875,955 MPa. For low pressures, the atom pairs associated with smaller shear rates ($\dot{\gamma }\leq 2\times 10^{8}\;s^{-1}$ for 0.1 MPa and $\dot{\gamma }\leq 2\times 10^{7}\;s^{-1}$ for 100 MPa) cluster together into compact groups that are not easily separable; for 0.1 MPa, these groups are vertically aligned. On the other hand, the atom pairs for higher shear rates are dispersed and spread out horizontally. For high pressures P≥ 400 MPa, all dimension reduction plots look similar. In each case, the atom pairs associated with all rates share the same overall pattern: they are spread out horizontally, and are somewhat separable by the PC2 dimension. This separation, however, does not correlate in any significant way with changes in shear rate. These observations are similar to the ones made for the dimension reduction of intramolecular far long backbone-backbone pairs in Figure 12. Far long backbone-backbone pairs were also able to separate the orientation tensor datasets, but the well-separated clusters were not organized along strain rate. Overall, the PCA results show that shear thinning at high pressures is not related to changes in the orientation order associated with the intermolecular atom pairs.

**Figure 18:**
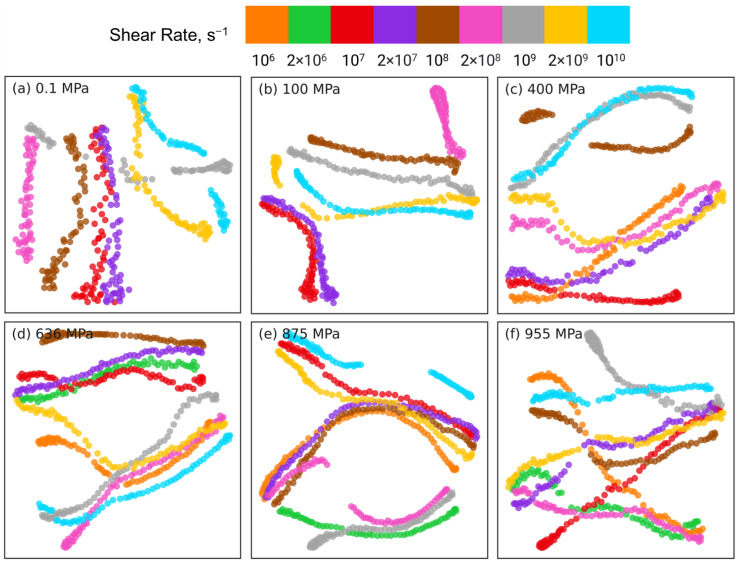
Dimension reduction using t-SNE of 61 intermolecular pair orientation tensors describing the intermolecular structure of squalane sheared at six different pressures: (**a**) 0.1 MPa, (**b**) 100 MPa, (**c**) 400 MPa, (**d**) 636 MPa, (**e**) 875 MPa, and (**f**) 955 MPa. Each point represents one pair orientation tensor. Different colors denote different shear rates as shown in the legend panel at the top.

Figure 18 shows the t-SNE dimension reduction results for the intermolecular atom pairs at different pressures. Unlike PCA, t-SNE is able to separate the atom pairs associated with different strain rates for all rates at the low pressures P=0.1,100 MPa. For all pressures, the atom pairs associated with the same strain rate distribute in a linear manner across the two-dimensional t-SNE plot. At low pressures, there is a degree of organization in the separation of different “strands” of atom pairs associated with the same shear rate. However, at high pressures, there is no consistent pattern in the organization of these strands, which is similar to the linear dimension reduction results obtained via PCA.

**Figure 19:**
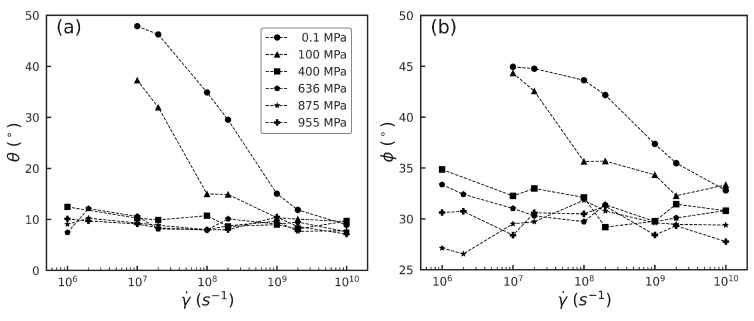
(**a**) Angle θ between the distance vector associated with the atom pair 2–28 and the flow direction $\hat{x}$ vs. strain rate $\dot{\gamma }$. (**b**) Angle ϕ between the distance vectors associated with the 2–28 atom pair belonging to two neighboring squalane molecules vs. $\dot{\gamma }$. Results shown for pressures listed in (**a**).

The PCA and t-SNE results suggest the saturation of the molecular orientation order associated with squalane sheared under different strain rates at high pressures. Motivated by this, in order to quantify the degree of evolution in the molecular orientation with shear rate for different pressures, we extract two angles, θ and ϕ, associated with the intramolecular and intermolecular orientation, respectively. We choose a far long backbone-backbone atom pair, 2–28, to compute the angle θ between the squalane molecule axis and the flow direction ($\hat{x}$). Figure 19a shows that at low pressure P≤ 100 MPa, θ decreases slowly from ∼$45^{{}^{\circ}}$ at strain rate $\dot{\gamma }=10^{7}$$s^{-1}$ to ∼10${}^{{}^{\circ}}$ at $\dot{\gamma }=10^{10}$$s^{-1}$, indicating that the squalane molecules gradually align along the flow direction with increasing shear rate. In contrast, for high pressures P≥ 400 MPa, θ is ≈$10^{{}^{\circ}}$ regardless of the strain rate, indicating that the squalane molecules are already well-aligned and the system exhibits a saturated intramolecular orientation order. These trends are consistent with the PCA and t-SNE dimension reduction results shown in Figure 12 and Figure 14.

Figure 19b shows the angle ϕ between two neighboring squalane molecules, which is computed as an average using the distance vectors associated with the 2–28 atom pair belonging to two neighboring squalane molecules. The average is taken over all pairs of squalane molecules whose center of masses are separated by a distance less than 10Å. Similar to the single molecule results, ϕ decreases with increasing $\dot{\gamma }$ at low P=0.1,100 MPa, varying from ∼$45^{{}^{\circ}}$ to ∼$33^{{}^{\circ}}$. On the other hand, at high P≥400 MPa, ϕ exhibits a much smaller variation with $\dot{\gamma }$, oscillating around ∼$30^{{}^{\circ}}$. Overall, these results indicate that shear thinning at low pressures is correlated with molecular alignment, however, at high pressures, it occurs with little change in the molecular structure as encapsulated by the intramolecular and intermolecular orientation tensors.

## 4. Conclusions

We have explored the potential of the NEMD-ML approach in probing the molecular-scale origins of the rheological properties of short-chain molecular liquids. The exploration is based on NEMD simulations of shear flow of squalane at 293 K over a broad range of strain rates $\dot{\gamma }\in 10^{6}–10^{10}$ $s^{-1}$ for which squalane exhibits substantial shear thinning for a wide range of pressures P∈0.1–955 MPa. ML is used to reduce and visualize the high-dimensional molecular orientation datasets generated by these simulations that encapsulate the molecular structure information.

Dimension reduction using PCA of intramolecular orientation tensor datasets at low pressures P=0.1,100 MPa showed that atom pairs, regardless of the pair type (side-backbone, end-backbone, short backbone-backbone, and long backbone-backbone pairs), are grouped together into well-separated clusters that are organized by strain rate along the PC1 axis. Dimension reduction of the side-backbone, end-backbone, short backbone-backbone, and long backbone-backbone pairs separately via PCA showed that the changes in the orientation of different types of pairs are correlated with the shear rate for these low pressures. These results bolster support for the use of power-law models such as the Carreau model for describing the flow curves in low-pressure (small Newtonian viscosity) conditions.

However, at high pressures P≥400 MPa, instead of strain rate, the atom pairs are grouped together and better separated by pair type. This signals an overall limited evolution of orientation of these atom pairs with changes in shear rate at high pressures. A closer examination of the long backbone-backbone pairs revealed that atom pairs separated by more than 16 bonds were able to separate into different clusters by strain rate, but these clusters were distributed randomly across the 2D space formed by the two principal components. These PCA findings were cross-checked by the t-SNE dimension reduction results. Overall, no clear evidence to link shear thinning at high pressures with rate-dependent changes in intramolecular orientation was found.

To further explore the correlation between shear thinning and molecular structure at high pressures, the orientation tensors of intermolecular atom pairs were calculated for different separation distances within 3.9–10 Å. Dimension reduction of these intermolecular orientation tensor datasets using PCA revealed a similar contrast between the molecular structure at low pressures versus that at high pressures. For P≥ 400 MPa, while the atom pairs grouped into well-separated clusters, no clear organization with respect to shear rate was observed, indicating that shear thinning is not related to changes in the intermolecular orientation with strain rate. t-SNE results corroborated many of the PCA findings. The saturation of the molecular orientation at high pressures was reaffirmed by extracting angular metrics that quantify the alignment of squalane molecules. The intramolecular alignment angle was observed to be ∼$10^{{}^{\circ}}$ with respect to the flow direction, and the angle between neighboring squalane molecules was saturated at ∼$30^{{}^{\circ}}$ over the entire range of strain rates during which squalane underwent substantial shear thinning.

Overall, dimension reduction results showed that shear thinning of squalane at high pressures does not rely on changes in intramolecular and intermolecular order, and therefore the viscosity reduction with increasing rate is likely the result of alternate mechanisms such as thermal-activation based flow processes, which fit the macroscopic flow curves data very well. We realize that we have explored only one liquid, so our conclusions are not generalizable to other fluids. However, the NEMD-ML approach developed in this work is versatile and can be used to investigate a wide variety of fluids. Extension of the NEMD-ML approach to probe the microscopic origins of the flow properties of other small-molecular fluids and long-chain polymeric liquids will be a subject of future work.

## Figures and Tables

**Table 1:** Lennard-Jones potential parameters.

United Atom	σ(Å)	ϵ(kcal/mol)
${\mathrm{CH}}_{3}$	3.930	0.227
${\mathrm{CH}}_{2}$	3.930	0.093
CH	3.810	0.080

**Table 2:** Dihedral potential parameters.

	$X-{\mathrm{CH}}_{2}-{\mathrm{CH}}_{2}-Y$	$X-{\mathrm{CH}}_{2}-\mathrm{CH}-Y$
$A_{1}$	2.007	0.814
$A_{2}$	−4.012	−1.792
$A_{3}$	0.271	0.389
$A_{4}$	6.290	3.673
$A_{5}$	0.000	0.000

## Data Availability

Our code and the data used are publicly available on GitHub at https://github.com/softmaterialslab/mlflow/ (accessed on 28 April 2023).

## Abbreviations

The following abbreviations are used in this manuscript:

LAMMPS	Large-scale atomic/molecular massively parallel simulator
LJ	Lennard-Jones
ML	Machine learning
NEMD	Nonequilibrium molecular dynamics
PBCs	Periodic boundary conditions
PCA	Principal component analysis
t-SNE	t-distributed stochastic neighbor embedding
